# A regulatory perspective on the availability of medicines and medicine shortages in outpatient care: case Finland

**DOI:** 10.1007/s11096-019-00850-2

**Published:** 2019-06-07

**Authors:** Kati Sarnola, Johanna Linnolahti

**Affiliations:** 1 Finnish Medicines Agency, Kuopio, Finland; 2 Finnish Medicines Agency, Helsinki, Finland

**Keywords:** Medicine availability, Finland, Medicine shortage, Outpatient care, Regulatory perspective

## Abstract

The good availability of medicines and medicine shortages have become global issues during recent years. Medicine shortages are often affected by universal determinants, but the availability of medicines is also depending on national characteristics. This commentary aims to discuss the issue of the availability of medicines and medicine shortages in outpatient care in Finland, taking in consideration both national characteristics and global determinants that effect on the availability of medicines in other countries but Finland also. In this commentary, a regulatory approach is applied.

## Impacts on practice

- Both national characteristics and global determinants should be taken into consideration when discussing on the availability of medicines and medicine shortages.
- From the regulatory perspective, mandatory shortage notifications, adequate stocks throughout the whole distribution network, information sharing and collaboration between stakeholders and public involvement are important factors in mitigating the effects of shortages.

## Introduction

The good availability of medicines and medicine shortages have become global issues during recent years [1]. Medicine shortages are known to cause medical errors, serious adverse events and delays in medical care, but they also to add to patients’ distrust of healthcare professionals, which may indirectly affect patient care. Furthermore, shortages may increase the costs of medical care from both patients’ and societies’ perspectives. Even though shortages are a global issue that is affected by universal determinants, the availability of medicines is also reliant on national characteristics [2]. Understanding the nature of these characteristics and their impacts on medicine shortages may be helpful in mitigating shortages in the broader sense.

### The pharmaceutical market and distribution network

The pharmaceutical market in Finland is small, approximately 0.3% of the global pharmaceutical market and 1.2% of the European pharmaceutical market [3]. Due to the small size of the pharmaceutical market, representatives of pharmaceutical companies and pharmaceutical wholesalers consider that the volumes are small, and the market may appear unattractive [4]. This poses the risk of companies opting out of the market rather than trying to cope with limited volumes and profits. In the long run, this leads to a limited number of operating companies in the market and may thus complicate shortage issues.

Finland is heavily dependent on foreign manufacturing and imports [3]. Most of the medicines sold in Finland originate from other European Union (EU) countries. In recent decades, the global pharmaceutical market has been centralized and, on the other hand, fragmented in outsourced manufacturing processes: production is commonly centralized to big contract manufacturers that often outsource production and testing phases to other operators [4, 5]. These operators may be situated far from the markets in which the products are eventually sold. Furthermore, Finland’s dependence on foreign manufacturing, its limited number of operating companies and its long or complex production chain pose a risk of medicine shortages [4]. The operators that are committed to providing a safe and continuous supply of medicines are in a key position for securing patient treatments.

The distribution of medicines from pharmaceutical companies to wholesalers in Finland is based on a single-channel system [6]. In the practical sense, a pharmaceutical manufacturer and a wholesaler establish an exclusive distribution contract, and pharmacies can typically acquire a certain pharmaceutical product through that particular wholesaler. A single-channel system may cause medicine shortages if the single wholesaler faces major disruptions. Such disruptions occur rarely, but were experienced in Finland in September 2017 [7]. Another factor posing a risk to medicine distribution is that there are typically only a couple of full-line distributors in a country like Finland [8]. In the case of a disruption, other wholesalers may not be able to handle the vast amount of orders.

Medicines are distributed to the public in outpatient care via community and university pharmacies [9]. There is at least one pharmacy in almost every municipality. Furthermore, Finnish pharmacies operate with longer working hours in comparison to the pharmacies in other Nordic countries [10]. This indicates that the availability of pharmaceutical services appears adequate. Regardless of this, in a study conducted in 2013, 80% of the studied pharmacies reported suffering from medicine shortages daily or almost daily [2]. However, only a third of all shortage cases were reported to cause problems for the pharmacies. Mainly, problems were not caused because a substitutable product was available. This indicates that pricing and reimbursement regulation may have a significant impact on the availability of medicines and on mitigating the shortage issue.

### Price and reimbursement regulation in outpatient care

Decisions about which products are included in the reimbursement system and their wholesale prices and reimbursement categories are made by the Pharmaceuticals Pricing Board [11]. Those medicines are then reimbursed from National Health Insurance, a universal public insurance for all permanent residents [12]. The system is based on manufacturers’ applications for reimbursement status and a reasonable wholesale price. Mandatory generic substitution and a reference price system are in use [13, 14]. Regardless of which products are reimbursed, the prices of the medicines are the same in all pharmacies.

Pricing and reimbursement are major influencers of the market introduction of medicines used in outpatient care. Several sources show that the low pricing and a lack of reimbursement may lead to a lack of market attractiveness from the perspective of pharmaceutical companies, which may eventually lead to unavailability [see, e.g. [4, 15] ]. From the regulatory perspective, it is important to secure the availability of medicines and market introduction of medicines [16]. At the same time, medicines should be available at reasonable prices for society.

### Notification of shortages

**Fig. 1:**
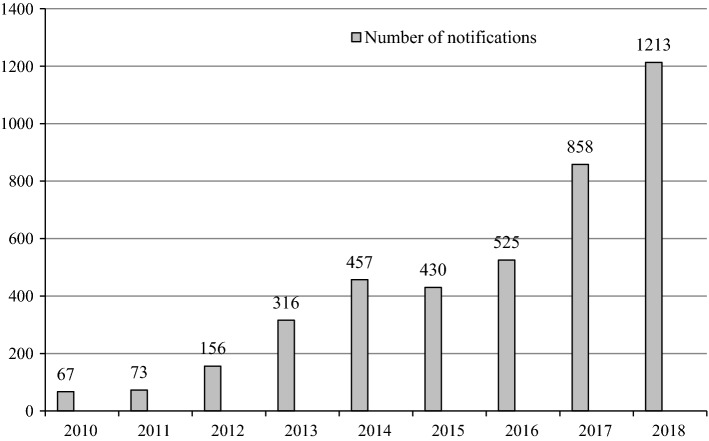
Shortage notifications from marketing authorisation holders to the Finnish Medicines Agency during 2010–2018

The reporting of medicine shortages in Finland is done through mandatory shortage notifications to the Finnish Medicines Agency from marketing authorisation holders [17]. Marketing authorisation holders are required to notify of shortages 2 months prior to the shortage unless there is an unforeseen quality defect or another exceptional circumstance that prevents them from doing so. The Finnish Medicines Agency publishes notifications. The number of shortage notifications sent to the Finnish Medicines Agency has increased extensively from 2010 (see Fig. 1).

The shortage notification system was originally developed to inform public and healthcare professionals of any shortages notified to the Finnish Medicines Agency. Due to the increased number of notifications, reporting no longer fills its original purpose. Therefore, the reporting will be part of the registry information of a medicinal product in the near future. This will later allow integrating the shortage information with other IT systems and databases, meaning that the information could be used by healthcare professionals in practical work (for example, it could be used by physicians when they prescribe medicines).

In order to make sure that the information that is shared by multiple IT systems is accurate and useful for healthcare professionals, the notification of shortages should be mandatory. Similarly to any other medicines’ legislation, sanctions for not being compliant with the legislation should be efficient and inspections of marketing authorisation holders should be possible. By these means competent authorities would be able to verify the accuracy of the provided information, if necessary.

### Adequate stocks throughout the whole distribution network

Mandatory reserve supplies are one of the means of tackling critical medicine shortages in Finland [18]. Storage varies from 3 to 10 months’ average supply, depending on the medicine and including critical and widely-used medicines. Manufacturers and wholesalers receive yearly compensation from the government for this storage. The compensation varies according to the product and is additionally dependent on the national interest rate of the Bank of Finland and the capital needed for the purchase. The costs associated with the mandatory reserve supplies are managed by the National Emergency Supply Agency (NESA) and financed by the extra-budgetary National Emergency Supply Fund [19].

**Fig. 2:**
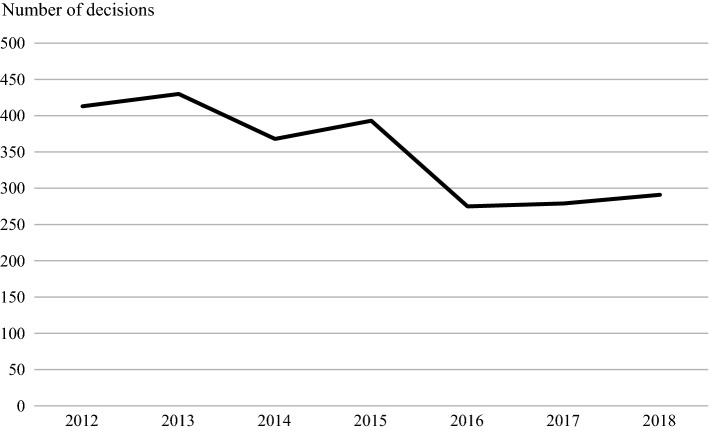
The number of decisions for exemption in order to maintain lower stock levels from 2012 to 2018

**Fig. 3:**
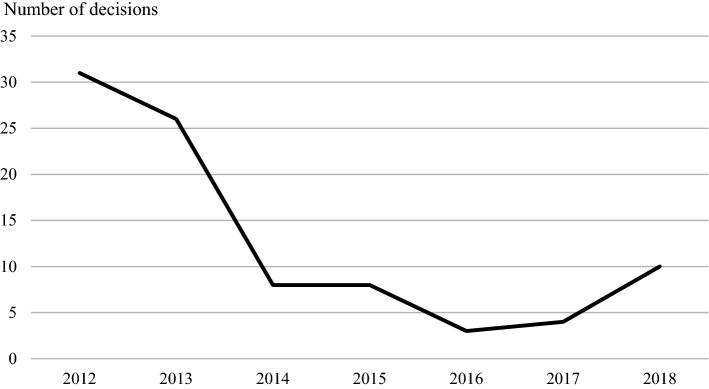
The number of decisions for exemption in order to maintain lower stock levels in the ATC category A10 from 2012 to 2018

Like any other national distribution systems, the mandatory reserve supply system is heavily dependent on the quality and continuous supply of medicines. Regardless of this, it has been proven to decrease the number of decisions made for exemption in order to maintain lower stock levels (Fig. 2), which indicates the mitigation of medicine shortages experienced later in the supply chain. The mitigation effect is especially clear in medicines that are used to treat diabetes (Fig. 3).

Furthermore, prior to a mandatory reserve supply or any other risk mitigation systems, it is essential that there is enough resilience towards disruptions in the whole distribution network. Manufacturers should make sure that there are enough stocks of raw materials and production capacity in multiple locations, as well as set minimum storage volumes in the distribution network that would help prevent patient impacts in the case of, for example, natural disasters. In addition, wholesalers and retailers should aim at stocking sufficient amount of alternatives in order to allow for any needed changes in the treatments in the case of major shortage issues. However, even a generic market may not be able to resolve shortage issues if there are recalls due to safety reasons and there are a very limited number of operators in the market.

### Topical global issues that may effect the national availability of medicines: falsified medicine regulation and Brexit

There are also topical issues that may affect the availability of medicines in the EU area and thus in Finland. In EU/EEA countries, medicines’ legislation for human medicinal products included new requirements for safety features for prescription medicines in order to prevent falsified medicines from penetrating the legal supply chain. Manufacturers implemented these changes in packaging and serialisation in IT systems by February 2019 in most EU/EEA countries (excluding Italy and Greece). In the worst-case scenario, missing data in the IT systems or system-related alerts could mean major disruptions throughout the whole supply chain when the product packages are being quarantined due to investigations of suspected falsification. So far, all operators of the supply chains have managed to mitigate shortages by taking the preventive measures agreed by all stakeholders into use. The effects on the overall availability of medicines are yet to be shown.

The withdrawal of the United Kingdom from the EU (Brexit) is expected to have an effect on the European Union pharmaceutical market in a number of ways. The United Kingdom is a major manufacturer inside the EU markets and there are also multiple marketing authorisation holders of medicinal products in the United Kingdom for the whole EU/EEA markets. After leaving the EU, all the marketing authorisation holders and manufacturing authorisation holders responsible for releasing products to EU/EEA markets, as well as certain qualified persons and safety master files, will have to be situated in the EU [20]. In the worst case, this would mean that a number of products might no longer be available and on the market due to the fact that EU requirements are not met in time for the withdrawal. Medicines regulatory authorities, as well as other governmental bodies, have made preparedness information on Brexit for all stakeholders and for the public in the other EU/EEA countries together with the European Commission [21].

### Information sharing and collaboration between stakeholders

Although national characteristics affect the availability of medicines, tackling the shortage issues can be done both nationally and multi-nationally. On the national level, modern communication systems should be developed in co-operation with the pharmaceutical industry, operators of the distribution chain, regulatory authorities and healthcare professionals in order to meet the needs for information on shortages. Current legislation in the European Union does not require the notification of public or healthcare professionals about shortages. At least in the case of critical shortages, the best practice would be to contact the necessary parties with efficient means of communication and to secure the continuity of the treatment of patients with safe medicinal products. From the patient perspective, unnecessary anxiety and harm are avoided when patients are notified of the shortage prior to their visits to pharmacies. Prescribing physicians may also require support, as switching stable medications may also cause anxiety for the prescribing physicians, especially when there are only poor alternatives to fill in for the medicines in shortage. Information on best practices may thus be vital.

On both national and multi-national levels, regulatory authorities in the EU and in the European Medicines Agency have worked towards compiling tools for the notification, coordination and assessment of the communication and reporting of medicine shortages. The task force set up by the Heads of Medicines Agencies and the European Medicines Agency has published a work programme for the years 2019–2020 in order for regulators and industry to ensure the availability of medicines for the benefit of patients in the EU. The key priorities include developing strategies to improve the prevention and management of shortages and encouraging best practices within the industry to prevent shortages [22]. In the global pharmaceutical market, the wider the co-operation between authorities and industry is, the better an understanding there is of the importance of the sharing and coordination of information. This coordination should include a multiple number of authorities even in a single country. In this sense, medicines regulators not only include licensing and inspecting bodies but also reimbursement and health technology assessment bodies and sometimes communal assessment bodies.

### Public involvement and patient perspective

Besides taking care of patients by securing the availability of medicines and sharing appropriate information, patients can and should be involved in more targeted and effective communication with the regulators. Although further research is needed in order to identify the impact of patient and public involvement [23], patient and consumer advisory boards have shown potential for promoting patient-centred practice improvements [24]. In Sweden, for example, a Patient and Consumer Advisory Board has been set up to increase patients’ insight into regulatory activities and responsibilities by, for example, providing their comments on the information texts issued to the public [25]. From the regulatory perspective, patient and public involvement may, for example, increase the intelligibility and legibility of the materials provided by the regulatory body or provide important viewpoints for authorities to take into account when securing patient access to medicines.

In addition, in the case of medicine shortages, public involvement may be one of ways of sharing information. Patient associations sometimes provide a forum for regulatory authorities and industry to share information about shortages of critical medicinal products bearing in mind any restrictions on informing the public about prescription medicines. With timely information on the shortage from the patient organisation or another forum, patients are then able to contact prescribing physicians well before their medicines run out. Information sharing is especially important in the case of shortages of essential medicines, such as orphan medicines.

## Conclusions

Similarly to any other country, both national characteristics and global determinants affect the availability of medicines and the occurrence of medicine shortages in Finland. From the regulatory perspective, mandatory shortage notifications, adequate stocks throughout the whole distribution network, information sharing and collaboration between stakeholders and public involvement are important factors in mitigating the effects of shortages.

## References

[1] Besancon L, Chaar B. Report of the international summit on medicines shortage 2013. Final report. Toronto (CA): International Pharmaceutical Federation; 2013; 24p.

[2] Heiskanen K, Ahonen R, Karttunen P, Kanerva R, Timonen J (2015). Medicine shortages—a study of community pharmacies in Finland. Health Policy.

[3] European Federation of Pharmaceutical Industries and Associations. The Pharmaceutical industry figures - Key Data 2018. Final Report. Brussels (BE): European Federation of Pharmaceutical Industries and Associations 2018; 28p.

[4] Heiskanen K, Ahonen R, Kanerva R, Karttunen P, Timonen J (2017). The reasons behind medicine shortages from the perspective of pharmaceutical companies and pharmaceutical wholesalers in Finland. PLoS ONE.

[5] Canadian Pharmacists Association. Canadian drug shortages survey. Final Report; 2010.

[6] Pelkonen E. Ajankohtaista Suomen lääkejakelusta. [Current topics in Finnish pharmaceutical distribution]. In: Hanhela. Lääkkeistä terveyttä? Helsinki: Lääketietokeskus; 2010. p. 28–30.

[7] Oriola Corporation. Helsinki: Oriola Corporation; 2017. www.oriola.com/publications/stock-exchange-releases/2017/oriola-corporations-third-quarter-result-impacted-by-distribution-disruptions-in-finlan. Accessed 12 April 2019.

[8] Pharma Industry Finland. Helsinki: Pharma Industry Finland; 2019. http://www.pif.fi/en/medicine/medicine-distribution. Accessed 12 April 2019.

[9] The Association of Finnish Pharmacies. Helsinki: The Association of Finnish Pharmacies; 2018. www.apteekkariliitto.fi/media/3-apteekkariliitto.fi/englanti/annual-reviews/annual_report_2017.pdf. Accessed 28 March 2019.

[10] The Association of Finnish Pharmacies. Helsinki: The Association of Finnish Pharmacies; 2013. www.apteekkariliitto.fi/media/pdf/afp_annual_review_2012_web.pdf. Accessed 28 March 2019.

[11] Pharmaceuticals Pricing Board. Helsinki: Pharmaceuticals Pricing Board; 2015. www.hila.fi/en/operations-and-organisation/tasks. Accessed 28 March 2019.

[12] Hakkarainen KM, Kivioja A, Saastamoinen LK, Babar Z-U-D (2015). Drug prices in Finland. Pharmaceutical prices in the 21st century.

[13] Pharmaceuticals Pricing Board. Helsinki: Pharmaceuticals Pricing Board; 2015. www.hila.fi/en/reference-price-system. Accessed 28 March 2019.

[14] The Social Insurance Institution of Finland. Helsinki: The Social Insurance Institution of Finland; 2018. www.kela.fi/web/en/medicine-expenses-generic-substitution-and-the-reference-price-system?inheritRedirect=true. Accessed 28 March 2019.

[15] Morrison A. Drug supply disruptions [environmental scan issue 17]. Final Report. Canadian Agency for Drugs and Technologies in Health; 2011.

[16] Panteli D, Arickx F, Cleemput I, Dedet G, Eckhardt H, Fogarty E (2016). Pharmaceutical regulation in 15 European countries review. Health Syst Transit.

[17] Medicine Act 10.4.1987/395. Finlex Data Bak; 2019. www.finlex.fi/en/. Accessed 12 April 2019.

[18] Finnish Medicines Agency. Helsinki: Finnish Medicines Agency; 2019. www.fimea.fi/web/en/supervision/mandatory_reserve_supplies. Accessed 28 March 2019.

[19] National Emergency Supply Agency. Helsinki: National Emergency Supply Agency; 2019. www.nesa.fi/organisation/funding-and-legislation/funding-the-security-of-supply/. Accessed 17 April 2019.

[20] European Commission and European Medicines Agency. Questions and answers related to the United Kingdom’s withdrawal from the European Union with regard to the medicinal products for human and veterinary use within the framework of the Centralised Procedure. Rev 04, 1 February 2019.

[21] European Commission. European Union; 2019. https://ec.europa.eu/info/brexit/brexit-preparedness_en. Accessed 12 April 2019.

[22] Heads of Medicines Agencies and the European Medicines Agency. Heads of Medicines Agencies and the European Medicines Agency; 2019. www.hma.eu/fileadmin/dateien/HMA_joint/00-_About_HMA/03-Working_Groups/TF_Availability/2018_08_TF_AAM_Press_release_Towards_Availability.pdf. Accessed 12 April 2019.

[23] Gamble C, Dudley L, Allam A, Bell P, Goodrare H, Hanley B (2014). Patient and public involvement in the early stages of clinical trial development: a systematic cohort investigation. BMJ Open.

[24] Sharma AE, Willard-Grace R, Willis A, Zieve O, Dubé K, Parker C (2016). How can we talk about patient-centered care without patients at the table? Lessons learned from patient advisory councils. J Am Board Fam Med.

[25] Sjöström B. Sweden: Swedish Medicines Agency’s Patient- and Consumer Advisory Board; 2018. https://lakemedelsverket.se/upload/ovrigt/MPA_Patient-and-Consumer-Advisoy-Board-B-Sjostrom-LV-2.pdf. Accessed 28 March 2019.

